# Postprandial glucose-lowering effects of fermented red ginseng in subjects with impaired fasting glucose or type 2 diabetes: a randomized, double-blind, placebo-controlled clinical trial

**DOI:** 10.1186/1472-6882-14-237

**Published:** 2014-07-11

**Authors:** Mi-Ra Oh, Soo-Hyun Park, Sun-Young Kim, Hyang-Im Back, Min-Gul Kim, Ji-Young Jeon, Ki-Chan Ha, Won-Taek Na, Youn-Soo Cha, Byung-Hyun Park, Tae-sun Park, Soo-Wan Chae

**Affiliations:** 1 Clinical Trial Center for Functional Food, Chonbuk National University Hospital, 20 Geonji-ro, Jeonju, Jeonbuk 561-712, Republic of Korea; 2 Clinical Trial Center, Chonbuk National University Hospital, 20 Geonji-ro, Jeonju, Jeonbuk 561-712 Republic of Korea; 3 Healthcare Claims & Management Incorporation, 4-7 Gosapyeong 4-gil, Jeonju, Jeonbuk 560-822, Republic of Korea; 4 Wonkwang Pharmaceutical Co., Ltd., 165 Seokamro 11-gil, Chunpomyun, Iksan-si, Jeonbuk 570-952, Republic of Korea; 5 Department of Food Science and Human Nutrition, 567 Baekje-daero, Jeonju, Jeonbuk 561-756, Republic of Korea; 6 Department of Biochemistry, Chonbuk National University Medical School, 567 Baekje-daero, Jeonju, Jeonbuk 561-756, Republic of Korea; 7 Department of Endocrinology and Metabolism, Chonuk National University Medical School, 567 Baekje-daero, Jeonju, Jeonbuk 561-756, Republic of Korea; 8 Department of Pharmacology, Chonuk National University Medical School, 567 Baekje-daero, Jeonju, Jeonbuk 561-756, Republic of Korea

**Keywords:** Fermented red ginseng, Type 2 diabetes, Impaired fasting glucose, Postprandial glucose

## Abstract

**Background:**

Red ginseng is prepared by steaming raw ginseng, a process believed to increase the pharmacological efficacy. Further bioconversion of red ginseng through fermentation is known to increase its intestinal absorption and bioactivity, and bioconversion diminishes the toxicity of red ginseng’s metabolite. This study was conducted to investigate the effects of daily supplementation with fermented red ginseng (FRG) on glycemic status in subjects with impaired fasting glucose or type 2 diabetes.

**Methods:**

This study was a four-week long, randomized, double-blind, placebo-controlled trial. Forty-two subjects with impaired fasting glucose or type 2 diabetes were randomly allocated to two groups assigned to consume either the placebo or fermented red ginseng (FRG) three times per day for four weeks. Fasting and postprandial glucose profiles during meal tolerance tests were assessed before and after the intervention.

**Results:**

FRG supplementation led to a significant reduction in postprandial glucose levels and led to an increase in postprandial insulin levels compared to the placebo group. There was a consistently significant improvement in the glucose area under the curve (AUC) in the FRG group. However, fasting glucose, insulin, and lipid profiles were not different from the placebo group.

**Conclusion:**

Daily supplementation with FRG lowered postprandial glucose levels in subjects with impaired fasting glucose or type 2 diabetes.

**Trial registration:**

ClinicalTrials.gov: NCT01826409

## Background

Type 2 diabetes is the fastest-growing metabolic disorder in many parts of the world. The incidence of type 2 diabetes in 2000 was ~171 million worldwide, and the number is estimated to double by 2020 [1,2]. Type 2 diabetes patients have a higher risk of cardiovascular disease, and recent studies have also suggested that impaired fasting glucose and impaired glucose tolerance are risk factors for cardiovascular disease independent of type 2 diabetes [3]. Therefore, patients with impaired fasting glucose or impaired glucose tolerance have become an important target group for the primary prevention of cardiovascular disease.

Ginseng, which is derived from the root of Panax ginseng C.A. Meyer, has been used for centuries in health-promoting supplements in Asia. The crude ginseng extract exhibits many bioactivities, including anticancer, antidiabetic, anti-inflammatory, antioxidant, and immunomodulatory effects [4-7]. When ginseng is steamed before drying, it is called red ginseng, and its bioactivity seems to offer more potential uses than the unprocessed white ginseng roots [8]. The bioactive constituents of red ginseng include various saponins (ginsenosides, a group of triterpene glycosides) and non-saponins, with the pharmacological activity of red ginseng attributable mainly to these ginsenosides [9,10]. To date, 50 ginsenosides have been identified in red ginseng. In the gastrointestinal tract, these ginsenosides are further metabolized by intestinal microorganisms, which improves their intestinal absorption, increases bioactivity, and diminishes the toxicity of the metabolite [11,12].

According to the literature, fermentation using microorganisms for the production of more effective compounds has been extensively studied. In particular, the pharmacological effects of a new saponin generated by red ginseng fermentation have been reported, and this saponin can be mass produced. Fermented red ginseng (FRG) can be metabolized by intestinal microorganisms, resulting in the transformation of ginsenosides Rb1, Rb2, Rc, and Rd into compound K, which is active in cancer, diabetes, and immune stimulation [13]. Also, fermentation of ginseng using lactic acid bacteria (Bifidobacterium sp., Lactobacillus sp., etc.) transforms Rg3 into Rh2, and this biotransformation increases cytotoxicity against tumor cells, potentiates an anti-allergic effect against mast cells, and exhibits anti-inflammatory activity [14-18]. These studies demonstrate that fermentation of red ginseng transforms its pharmacological ingredients into low molecular weight active compounds and results in greater absorption.

In recent years, studies have reported that fermentation increases the hypolipidemic and hypoglycemic effects of red ginseng [18], and FRG protects pancreatic β-cells from streptozotocin toxicity [19]. However, to our knowledge, functional studies regarding the anti-diabetic effects of FRG in humans have never been conducted. In this study, red ginseng was subjected to fermentation with Lactobacillus plantarum, and the postprandial glucose-lowering activity of FRG was investigated in subjects with impaired fasting glucose or type 2 diabetes.

## Methods

### Participants

The study subjects were recruited from May 2008 to August 2009 in the Clinical Trial Center for Functional Foods at Chonbuk National University Hospital via a local newspaper advertisement. A total of 42 healthy male and female subjects agreed to participate in the study. Subjects age 20–75 years with a fasting glucose of 5.6-7.8 mmol/l with at least two follow-up measurements were recruited and included in the study. Exclusion criteria for the study included the following: (1) a history of diabetes mellitus or glucose-lowering agent use or insulin therapy within three months prior to screening, (2) lipid metabolism disorders, (3) acute or chronic inflammatory disease, (4) corticosteroid medication use within weeks of the study, (5) cardiovascular disease (arrhythmia, heart failure, myocardial infarction, or patients with pacemakers), (6) an allergy or hypersensitivity to any of the ingredients in the test products, (7) a history of disease that could interfere with the test products or impede their absorption, such as gastrointestinal diseases (Crohn’s Disease) or gastrointestinal surgery (appendicitis or enterocele), (8) participation in other clinical trials within the previous two months, (9) abnormal hepatic liver function or renal disease (acute/chronic renal failure or nephrotic syndrome), (10) use of medication containing lipid phosphates within three months of the study, (11) use of anti-psychosis drug therapy within two months of the study, (12) laboratory tests, medical, or psychological conditions deemed by the investigators to interfere with successful participation in the study, (13) a history of alcohol or substance abuse, and (14) pregnancy or current breast feeding. All subjects gave their written, informed consent before entering the study. The study, which was conducted according to the Declaration of Helsinki, was approved by the Functional Food Institutional Review Board (FFIRB) of Chonbuk National University Hospital (WKP-FG7070-001).

### Preparation of FRG

Fermented red ginseng extract was provided by WON PHARM Co., Ltd. (Iksan, Korea). The red ginseng was prepared by steaming the root of Panax ginseng C.A. Meyer (at least four-year-old white ginseng) at 98-100 for 4 h and drying the ginseng at 60°C for 5 h. Next, the fermented red ginseng was extracted with 60% ethyl alcohol at 70°C. The red ginseng extract was suspended in water, fermented for 15 days by previously cultured *Lactobacillus plantarum* at 35-40°C, and then freeze-dried. Ginsenoside profiles in the FRG were analyzed by high performance liquid chromatography (HPLC) according to Korean Food and Drug Administration (KFDA) guidelines, and these ginsenoside profiles are shown in Table 1. The placebo was composed primarily of dried yeast, and the flavor, energy content, appearance, and dosage were matched.

**Table 1:** Ginsenoside profile of the FRG

Ginsenoside*	**Red ginseng (%/g)**	**FRG (%/g)**
Rg1	0.30	0.12
Re	0.30	0.35
Rb1	0.65	0.33
Rb2 + Rc	0.28	0.50
Rd	0.02	0.24
Rg3	0.09	0.19
Rh2	0.08	0.13
Compound K	0.05	0.49
Total	1.77	2.35

^*^Determined by high performance liquid chromatography (HPLC).

### Study design

This study was a four-week long, randomized, double-blind, placebo-controlled clinical trial, performed according to a computer-generated randomization schedule designed to assign subjects to the FRG or placebo groups. Neither the investigators nor the subjects knew the randomization code or the results of the blood glucose levels until after statistical analysis was complete. Subjects attended a screening visit (within two weeks), at which inclusion and exclusion criteria were assessed. The enrolled subjects were scheduled for their first visit, and subjects were randomly assigned to one of two groups, either the FRG (n = 21) or placebo group (n = 21). Subjects received either the FRG or placebo capsules every week, and all subjects were instructed to take either three FRG capsules or three placebo capsules per day (2.7 g/day) for four weeks.

Subjects were asked to visit the research center once every week for a total six visits, which included the screening visit (screening, 0, 1, 2, 3 and 4 weeks). After the subjects were screened, we performed a meal tolerance test. The subjects were asked to consume a standard meal [584.1 kcal, caloric contribution: 52% carbohydrates (containing 70 g of available carbohydrate), 18% protein, and 30% fat] after a 12-h overnight fast. To determine the postprandial glucose and insulin responses, venous blood samples were taken at 0, 30, 60, 90, and 120 min. The 0 min sample was used to determine fasting plasma glucose and insulin levels. Also, fasting blood samples were collected at each visit to measure laboratory parameters and the lipid profile (total cholesterol, HDL cholesterol, LDL cholesterol, and triglycerides). During the assessment period, subjects were instructed to sit quietly.

During the intervention period of four weeks, subjects were asked to continue their usual diets and activity and to not ingest any other functional foods or dietary supplements. Anthropometric and biochemical parameters, vital signs, and nutrient intake were measured before and after the intervention period. Every week, the subjects were asked to report any adverse events or changes in training, lifestyle, or eating pattern, and to assess capsule-dosing compliance.

A CONSORT checklist for the reporting of this study can be found in Additional file 1.

### Biochemical analyses

Blood samples were analyzed on a Hitachi 7600–110 analyzer (Hitachi High-Technologies Corporation, Japan). The total area under the curve (AUC) of the glucose response during the meal tolerance tests was determined using the trapezoid method.

Safety was assessed by adverse events, physical examination, vital signs, and laboratory parameters (hematology, biochemistry, and urinalysis). Finally, compliance was assessed by the number of returned capsules.

### Statistical analysis

All of the presented data are from the intention-to-treat population. The primary outcome (postprandial glucose) and the secondary outcomes (total cholesterol, HDL cholesterol, LDL cholesterol, and triglycerides) were used to compare the FRG group to the placebo group. Statistical analyses were performed using SAS version 9.2 for Windows (SAS Institute, Cary, NC, USA). For statistics, we used a mixed-effect model approach for intention-to-treat analysis with missing values [20]. Data are shown as the mean values and the standard error of the mean (SEM). The sample size was statistically determined to obtain a power of 80% with an alpha of 0.05. In order to demonstrate effects in 2-h postprandial glucose level, which was calculated to be a 0.4 mmol/l reduction with a standard deviation of 0.53 mmol/l, a sample size of 32 (16 in the FRG group and 16 in the placebo group) was required. Assuming a 20% loss to follow-up, 42 participants were selected. The two groups were equal in size in order to obtain the greatest statistical power. General characteristics were analyzed by independent *t*-tests or Chi-square tests. The significance of the differences within or between groups were tested by a linear mixed-effect model and a paired *t*-test of the mean. Chi-square tests were performed to determine differences in the frequencies of categorized variables between groups. A value of p < 0.05 was considered statistically significant.

## Results

### Participant characteristics

**Figure 1:**
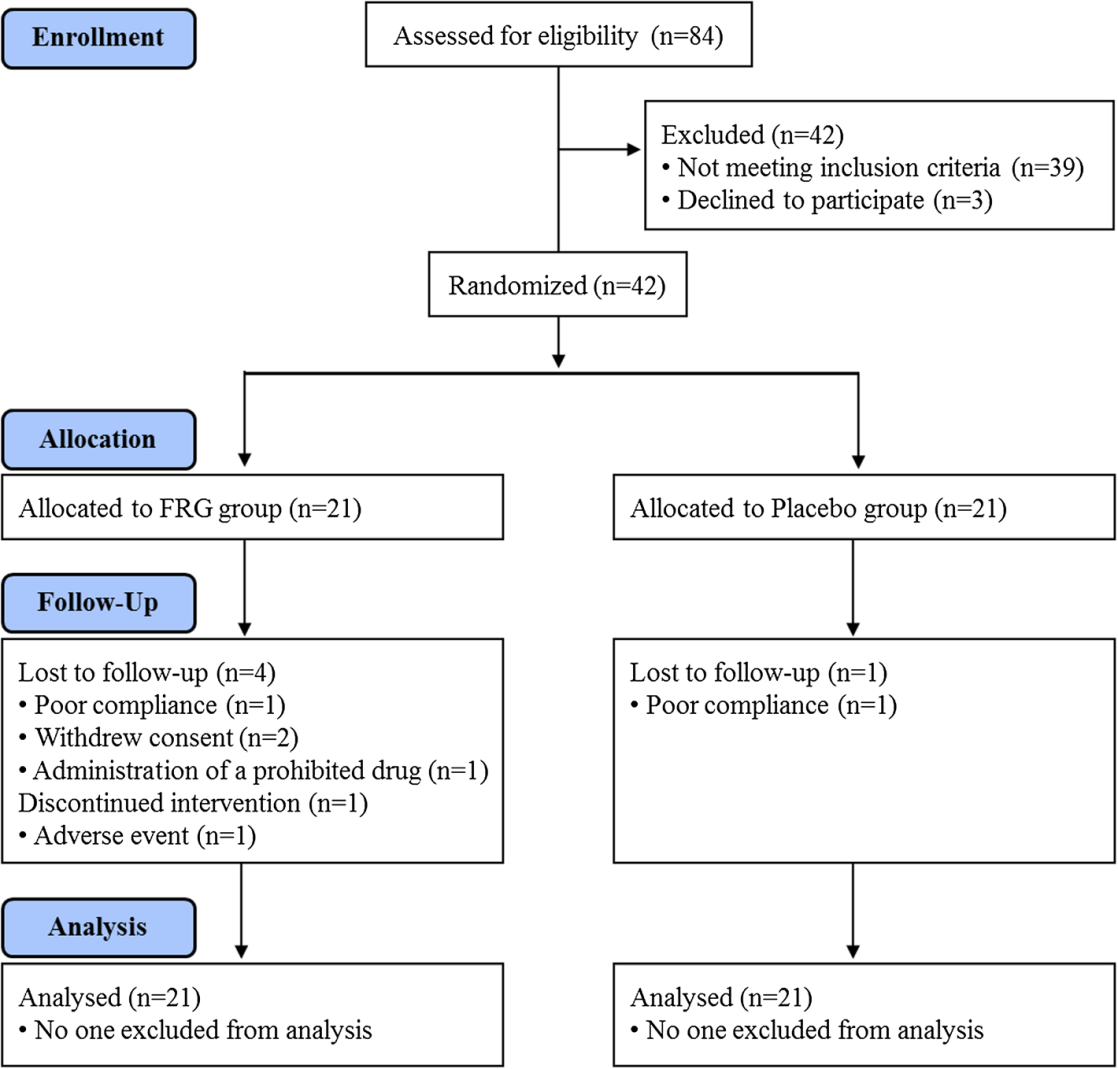
**Disposition of participants in the clinical trial of FRG versus placebo.** Number of study participants enrolled, allocated, followed, and analyzed, shown using the CONSORT 2010 Flow Diagram.

Eighty-four participants were assessed for eligibility, and a total of 42 participants (mean age = 53.3 ± 8.4 years, 28 males and 14 females, mean body mass index = 24.9 ± 3.3 kg/m^2^, fasting glucose = 6.5 ± 0.5 mmol/l) met the inclusion criteria. Nineteen participants were diagnosed with type 2 diabetes, and 23 participants had either impaired fasting glucose or impaired glucose tolerance. Among these participants, six dropped out for reasons which included lack of compliance (n = 2), administration of a prohibited drug (n = 1), withdrawal of consent (n = 2), and an adverse event (n = 1). Thus, a total of 36 participants completed the study (Figure 1). There were no significant differences between the two groups in age, gender, height, weight, body mass index, or fasting glucose levels (Table 2). The compliance rates, which were based on pill count, were 95.6 ± 4.8% and 95.4 ± 5.1% in the placebo and FRG groups, respectively. The diabetic participants did not receive antihyperglycemic medication or insulin therapy, and no participants with impaired fasting glucose or impaired glucose tolerance were newly diagnosed with type 2 diabetes during the study.

**Table 2:** Study participant demographic characteristics

	**Placebo group (n = 21)**	**FRG group (n = 21)**	***p-*** **value**^**1)**^
Sex (M/F)	15/6	13/8	0.513^2)^
Age (years)	53.5 ± 1.9	53.2 ± 1.8	0.914
Height (cm)	165.0 ± 1.8	166.6 ± 1.4	0.477
Weight (kg)	67.5 ± 2.4	68.9 ± 1.8	0.646
Waist (cm)	91.1 ± 1.7	93.0 ± 1.3	0.406
BMI (kg/m^2^)	24.9 ± 0.8	24.9 ± 0.7	1.000
Fasting plasma glucose (mmol/l)	6.4 ± 0.6	6.5 ± 0.5	0.737
Total cholesterol (mmol/l)	4.8 ± 0.1	5.2 ± 0.2	0.121
LDL cholesterol (mmol/l)	2.9 ± 0.1	3.1 ± 0.1	0.414
HDL cholesterol (mmol/l)	1.2 ± 0.1	1.3 ± 0.1	0.170
Triglycerides (mmol/l)	1.7 ± 0.1	1.7 ± 0.1	0.928

Data are presented as mean ± SEM. ^1)^Analyzed by independent *t*-tests and the *p*-value represents the comparison to the placebo group. ^2)^Analyzed by Chi-square tests. BMI: body mass index.

### Safety

At each visit, information about symptoms or side effects was recorded, but no severe side effects or serious adverse events were reported during the four-week study period. However, hypoglycemia was reported in the FRG group, which led to one subject withdrawing from the study.

### Meal tolerance test

**Figure 2:**
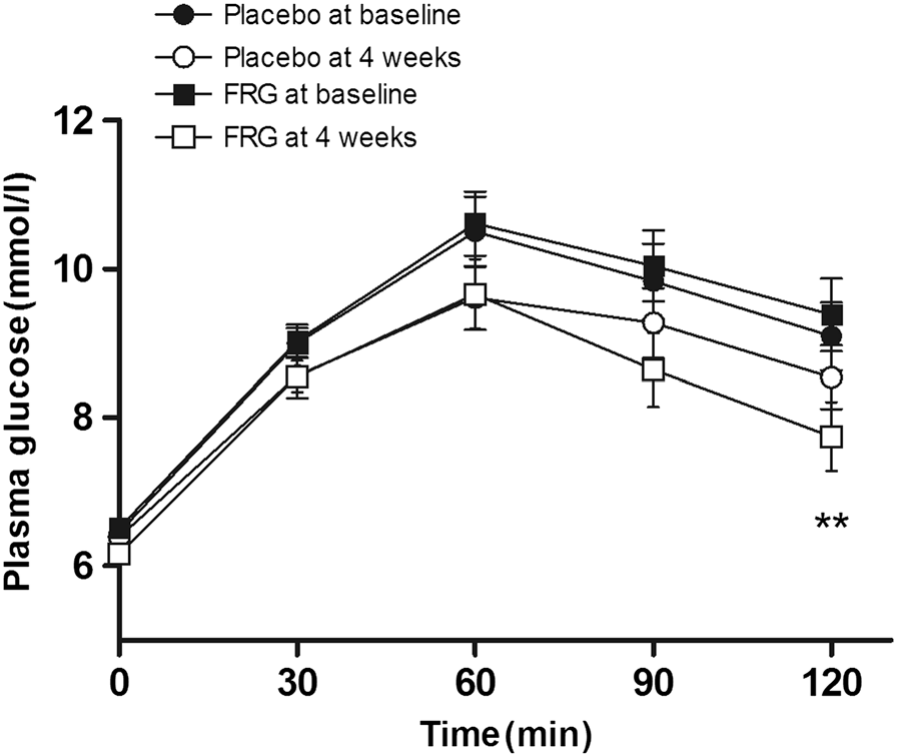
**The effect of FRG supplementation on glucose levels during meal tolerance tests.** Data are shown as mean ± SEM for 21 subjects. **p < 0.01 vs. placebo at four weeks.

The effects of FRG supplementation on postprandial glycemic variables were evaluated during a standardized breakfast test meal. Compared to the placebo, FRG supplementation significantly reduced the 2-h postprandial glucose level and increased the 2-h postprandial insulin level (Table 3). The fasting plasma glucose level was significantly reduced by FRG supplementation (*p* = 0.039), but did not show a treatment effect when compared to the placebo group. No differences in the fasting insulin level were found. Figure 2 shows the time-course changes in the plasma glucose levels during the meal tolerance test. Consistent with the significant change in the 2-h postprandial glucose level (*p* = 0.008), there was a significant difference in the AUC of the glucose response between the placebo and FRG groups (Table 3).

**Table 3:** Effect of FRG supplementation on postprandial glucose and insulin levels

	**Placebo group (n = 21)**			**FRG group (n = 21)**			***p-*** **value**^**2)**^
	**Baseline**	**Week 4**	***p-*** **value**^**1)**^	**Baseline**	**Week 4**	***p-*** **value**^**1)**^	
Fasting plasma glucose (mmol/l)	6.4 ± 0.61	6.3 ± 0.6	NS	6.5 ± 0.5	6.1 ± 0.6	0.039	NS
Postprandial plasma glucose_2h_ (mmol/l)	9.0 ± 2.1	8.5 ± 1.9	NS	9.3 ± 2.2	7.7 ± 1.9	0.0001	0.008
AUC_glucose_ (mmol/l)	18.4 ± 0.7	17.3 ± 0.7	0.045	18.6 ± 0.7	13.5 ± 1.6	0.002	0.013
Fasting plasma insulin (μU/ml)	7.4 ± 0.9	6.9 ± 0.6	NS	7.3 ± 1.0	9.0 ± 1.3	NS	NS
Postprandial plasma insulin_2h_ (μU/ml)	38.1 ± 3.9	35.5 ± 4.1	NS	39.2 ± 5.6	56.3 ± 9.8	NS	0.040

^1)^Analyzed by linear mixed-effect model and the *p*-value represents the comparison to the baseline visit. ^2)^Analyzed by linear mixed-effect model and the *p*-value represents the comparison to the placebo group. Data are presented as the mean ± SEM.

### Lipid profile changes

After four weeks of supplementation, there were no significant differences in total cholesterol, HDL-cholesterol, LDL-cholesterol, or triglyceride levels between the FRG and placebo groups (Table 4).

**Table 4:** Effect of FRG supplementation on lipid levels

	**Placebo group (n = 21)**			**FRG group (n = 21)**			***p-*** **value**^**2)**^
	**Baseline**	**Week 4**	***p-*** **value**^**1)**^	**Baseline**	**Week 4**	***p-*** **value**^**1)**^	
Total cholesterol (mmol/l)	4.9 ± 0.2	4.8 ± 0.2	NS	5.4 ± 0.2	4.9 ± 0.2	0.008	NS
HDL cholesterol (mmol/l)	1.2 ± 0.1	1.1 ± 0.1	0.022	1.3 ± 0.1	1.2 ± 0.1	0.014	NS
LDL cholesterol (mmol/l)	3.1 ± 0.2	2.8 ± 0.2	0.005	3.3 ± 0.2	2.9 ± 0.2	0.025	NS
Triglycerides (mmol/l)	1.6 ± 0.2	1.7 ± 0.2	NS	1.6 ± 0.2	1.9 ± 0.2	NS	NS

^1)^Analyzed by linear mixed effect model and *p*-value represents the comparison to the baseline visit. ^2)^Analyzed by linear mixed effect model and *p*-value represents the comparison to the placebo group. Data are presented as mean ± SEM.

## Discussion

Over the past few decades, various plant extracts have been clinically evaluated for the treatment of type 2 diabetes, including red ginseng, which has been shown to possess antidiabetic activity in cell cultures, animal studies, as well as in clinical studies [21-23]. To enhance the pharmacological efficacy of red ginseng, bioconversion using intestinal bacteria has been successfully implemented. During bacterial fermentation, ginsenosides are transformed into their readily absorbable and more potent deglycosylated forms. Because of the benefits this transformation confers, several intestinal bacteria have been used to ferment red ginseng, and the selection of an appropriate bacterial strain has become an essential prerequisite in achieving red ginseng’s desired pharmacological efficacy. In this study, we used L. plantarum for the microbial conversion of ginsenosides in red ginseng. L. plantarum has been traditionally used for kimchi fermentation and is considered to be a food-grade microorganism [24]. Using this bacteria, Kim et al. [25] found that fermented red ginseng contains a much higher concentration of total ginsenosides and metabolites, especially Rh1, Rh2, Rg5, Rk1, Rg2, Rg3, and compound K. Of note, FRG has enhanced immunological activities compared to red ginseng [26]. However, no studies have been conducted in which fermented red ginseng was used to treat diabetic patients.

The rationale for FRG supplementation in patients with impaired fasting glucose or type 2 diabetes is based on several reports. Ginseng is effective in the prevention of diabetes development in high-risk patients, and ginseng also improves glycemic control in patients with type 2 diabetes [27]. Notably, red ginseng supplementation reduces postprandial glycemia and improves symptoms in patients with type 2 diabetes [28,29]. Pharmacokinetic analysis of ginsenoside metabolites demonstrates a greater absorption rate of compound K in fermented ginseng than in non-fermented ginseng [30]. Also, fermentation of red ginseng berries with L. plantarum improves blood glucose parameters in diabetic db/db mice [31]. These studies suggest that fermentation may potentiate the action of red ginseng on the insulin-secreting pancreatic β-cells and the target tissues that take up glucose.

Our finding of reduced postprandial glucose levels and increased insulin levels in the FRG group during the meal tolerance test indicates an improvement in the glucose-stimulated insulin response. FRG’s beneficial effect on postprandial glucose concentration may be due to its direct effects on glucose absorption into the portal circulation [32,33], by improving insulin secretion from the pancreatic β-cells [34], or by removing glucose from the systemic circulation. Additionally, FRG could act indirectly by decreasing body weight or by lowering the plasma levels of toxic molecules such as oxygen free radicals and free fatty acids. Despite the lack of sufficient mechanistic studies, our findings of an improved glycemic response in the FRG group are consistent with those of Trinh et al. [18], who demonstrated improved glycemic responses in hypertriglyceridemic mice following supplementation with Bifidobacterium-fermented red ginseng. In contrast with our results, a recent human study reported that ginseng or ginsenoside therapy does not improve β-cell function or insulin sensitivity [35], suggesting that minimal bioavailability after oral ingestion limits red ginseng’s therapeutic efficacy in humans [35]. Given these results, in general, fermented red ginseng has more potent pharmacological activities than non-fermented red ginseng, and supplementation of FRG is beneficial for both type 2 diabetic patients and high-risk subjects with persistent impaired fasting glucose, at least in terms of stabilizing postprandial glycemia.

In this study, fasting plasma glucose levels, insulin levels, and lipid profiles were not different between the two study groups. In most human studies, improved fasting glycemia is expected over the long-term, whereas postprandial glycemia has been known to be more sensitive to small perturbations in glycemic control [36]. In addition, ginseng has been reported to improve blood lipid profiles. Studies by Kim et al. [34] and Yamamoto et al. [34] showed that high-dose (4.5 g/day) or long-term (eight weeks) ginseng supplementation improves dyslipidemia, decreases triglycerides, and increases HDL cholesterol. In this study, we supplemented subjects with a relatively lower dose (2.7 g/day) for a shorter period of time (four weeks), which might explain the lack of significant changes in fasting glucose, insulin, and the lipid profile.

The limitations of this study should be considered. First, this study was limited to a small number of subjects. Therefore, caution should be used in generalizing these results to other populations. However, these results are generally consistent with other studies reporting decreasing blood glucose responses and increasing insulin levels. Second, this study was only four weeks in duration, and thus, we only evaluated the short-term effects of FRG supplementation on our study outcomes. Finally, this study was a comparison study with a placebo group, wherein the psychological effects of the placebo and the effects of the placebo components were not considered.

In conclusion, for the first time, the results of this study demonstrated that FRG supplementation has the potential to improve postprandial plasma glucose levels and to increase insulin levels. These results suggest that FRG supplementation may be beneficial for individuals with impaired fasting glucose or type 2 diabetes. Further studies with a larger number of subjects over a longer duration are needed.

## Conclusion

In conclusion, the results of this study demonstrated for the first time that FRG supplementation has the potential to improve postprandial plasma glucose levels and to increase insulin levels. These results suggest that FRG supplementation may be beneficial for individuals with impaired fasting glucose or type 2 diabetes. Further studies with a larger number of subjects over a longer duration and comparative studies between fermented red ginseng and non-fermented red ginseng are needed.

## Abbreviations

FRG: Fermented red ginseng; AUC: Area under the curve; FFIRB: Functional food institutional review board; SE: Standard errors; SEM: Standard error of mean.

## Competing interest

Wonkwang Pharmaceutical Co., Ltd. gave us permission to use their commercially available FRG product for our study. Furthermore, they provided the intervention products and financial support for the completion of the study. Wonkwang Pharmaceutical Co., Ltd. had no further methodological or any other input in design, execution, or reporting of this study. Wonkwang Pharmaceutical Co., Ltd. had no access to the study protocol, names of participating physicians, or raw study data. Before starting the study, it was agreed specifically that the study would be published regardless of the results.

## Authors’ contributions

SWC and TSP conceived the study concept and designed the experiments. MRO, SHP, SYK, and HIB performed the experiments. MRO, JYJ, KCH, WTN, MGK, and BHP analyzed and interpreted the data and wrote the manuscript. YSC and BHP helped draft the manuscript. SWC and TSP had primary responsibility for the final content. All authors read and approved the final manuscript. We are grateful for the continuous discussions and support from BHP.

## Pre-publication history

The pre-publication history for this paper can be accessed here:

http://www.biomedcentral.com/1472-6882/14/237/prepub

## Supplementary Material

Additional file 1 CONSORT 2010 checklist*. Click here for file
